# Physicochemical Properties of C-Type Starch from Root Tuber of *Apios fortunei* in Comparison with Maize, Potato, and Pea Starches

**DOI:** 10.3390/molecules23092132

**Published:** 2018-08-24

**Authors:** Juan Wang, Ke Guo, Xiaoxu Fan, Gongneng Feng, Cunxu Wei

**Affiliations:** 1 Key Laboratory of Crop Genetics and Physiology of Jiangsu Province/Key Laboratory of Plant Functional Genomics of the Ministry of Education, Yangzhou University, Yangzhou 225009, China; juanwang@yzu.edu.cn (J.W.); 18115657147@163.com (K.G.); m150777@yzu.edu.cn (X.F.); ffyalce@ycit.cn (G.F.); 2 Co-Innovation Center for Modern Production Technology of Grain Crops of Jiangsu Province/Joint International Research Laboratory of Agriculture & Agri-Product Safety of the Ministry of Education, Yangzhou University, Yangzhou 225009, China

**Keywords:** *Apios fortunei*, root tuber, C-type starch, structural properties, functional properties

## Abstract

The dry root tuber of *Apios fortunei* contained about 75% starch, indicating that it is an important starch resource. Starch displayed spherical, polygonal, and ellipsoidal granules with central hila. Granule sizes ranged from 3 to 30 μm with a 9.6 μm volume-weighted mean diameter. The starch had 35% apparent amylose content and exhibited C_A_-type crystalline structure with 25.9% relative crystallinity. The short-range ordered degree in the granule external region was approximately 0.65, and the lamellar thickness was approximately 9.6 nm. The swelling power and water solubility began to increase from 70 °C and reached 28.7 *g*/*g* and 10.8% at 95 °C. Starch had typical bimodal thermal curve in water with gelatinization temperatures from 61.8 to 83.9 °C. The 7% (*w*/*w*) starch-water slurry had peak, hot, breakdown, final, and setback viscosities of 1689, 1420, 269, 2103, and 683 mPa s, respectively. Rapidly digestible starch, slowly digestible starch, and resistant starch were 6.04%, 10.96%, and 83.00% in native starch; 83.16%, 15.23%, and 1.61% in gelatinized starch; and 78.13%, 17.88%, and 3.99% in retrograded starch, respectively. The above physicochemical properties of *A. fortunei* starch were compared with those of maize A-type starch, potato B-type starch, and pea C-type starch. The hierarchical cluster analysis based on starch structural and functional property parameters showed that *A. fortunei* and pea starches had similar physicochemical properties and were more related to maize starch than potato starch.

## 1. Introduction

Starch is synthesized in plastid and exists as semicrystalline granules in plants. It contains transient (also named as assimilatory) and reserve starches. The reserve starch (usually called starch) is synthesized and stored in plant storage tissues including seed, fruit, tuber, rhizome, corm, bulb, and some metamorphosis roots [1,2,3,4]. It not only provides humans and animals with nutrition and energy but is also widely utilized in food and non-food industries due to its abundant availability and low cost. Starches from different botany sources have different physicochemical properties, leading to their different applications [1,2,3]. Cereal endosperm, legume embryo, and some tubers are conventional starch resources and have been extensively studied and commercial used [2,5,6,7,8,9]. However, there is demand for new starches to be found for the development of food and non-food industries. Therefore, some nonconventional starch resources have been studied in some fruit kernels [3], tubers of *Arisaema* species [10], and kernels of *Trapa* species [11,12] in recent years.

Starch mainly consists of amylose and amylopectin. The amylose is a mixture of linear and poorly branched polyglucans, and the amylopectin is highly branched polyglucans. The amylopectin branch-chains form double helices, which are laterally packed to form crystalline lattice. The crystalline lattice has two crystalline structures of A- and B-type allomorphs in plants [13]. However, three types of plant starch (A-, B-, and C-type) are reported according to their X-ray diffraction (XRD) patterns. A-type starch contains only A-type allomorph, and B-type starch contains only B-type allomorph [14]. Compared with A- and B-type starches, the C-type starch is complex and contains both A- and B-type allomorphs. The different proportions of A- and B-type allomorphs further classify C-type starches into C_A_-(closer to A-type), C_C_- (typical C-type), and C_B_-type (closer to B-type) [15]. Recently, He and Wei [15] summarizes four distribution patterns of A- and B-type allomorphs in C-type starch. (1) A- and B-type allomorphs exist in the same granule with centric hilum and are distributed in the outer and inner regions of granule, respectively, such as pea C-type starch [16,17]. (2) A- and B-type allomorphs exist in the same granule with centric hilum but are distributed in the inner and outer regions of granule, such as C-starch from high-amylose rice TRS [18]. (3) A- and B-type allomorphs exist in the same granule with eccentric hilum and are distributed in the different regions of granules such as lotus rhizome C-type starch [19,20]. (4) A- and B-type allomorphs exist in the different granules, such as some high-amylose maize starches [21].

The different distribution patterns and proportions of A- and B-type allomorph have important effects on the physicochemical properties and applications of C-type starches [15,22], though previous studies find that A- and B-type allomorphs are distributed in the same granules of normal C-type starches [16,17,18,19,20,21,22]. Recently, a new C-type starch has been detected in root tuber of *Apios fortunei*, and its A- and B-type allomorphs are distributed in different starch granules. The B-type granules have significantly lower gelatinization temperature than A-type granules, but their morphology, size, and amylose content are similar [23]. To our knowledge, this is the first time the allomorph distribution pattern of normal C-type starch has been reported. However, its physicochemical properties have not been investigated and compared with conventional and commercial starches, which restricts its applications in the food industry.

In this study, starch was isolated from root tubers of *A. fortunei* and its morphology, structure, and functional properties were investigated and compared with those of maize A-type starch, potato B-type starch, and pea C-type starch. Our objective was to characterize the physicochemical properties of starch from *A. fortunei* and provide some information for its utilization in food industry.

## 2. Results and Discussion

### 2.1. Starch and Soluble Sugar Contents in Root Tuber of A. fortunei

*A. fortunei* had 75.1% starch and 7.6% soluble sugar in dry root tuber. Usually, plant storage tubers and roots have starch contents from 30% to 88% [5], legume seeds contain starch from 20% to 47% [9], and cereal seeds have starch contents over 65% [24]. In addition, the dry root tuber of *A. americana* contains 68% starch [25]. Therefore, compared with conventional and commercial starch resource of cereal, legume, tuber, and root crops, the high starch content in *Apios* root tuber indicated that it is an important resource of starch.

### 2.2. Morphology and Granule Sizes of Starch

**Figure 1:**
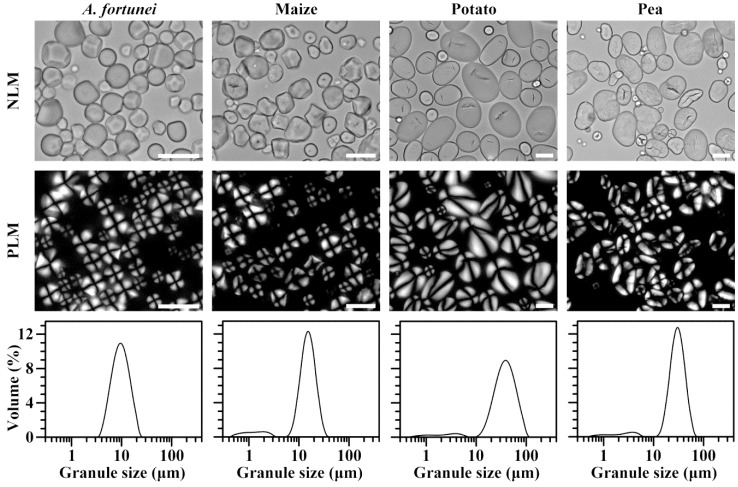
Morphology of starch under normal light microscope (NLM) and polarized light microscope (PLM) and granule size distribution. Scale bar = 20 μm.

The polarized light microscope was used to observe the morphology of starch granule under normal and polarized light. This result is present in Figure 1. *A. fortunei* starch had spherical, polygonal, and ellipsoidal shapes with central hila. Most starch granules of maize were polygonal with central hila; potato starch had small spherical granules with central hila and large ellipsoidal granules with eccentric hila; and most of pea starch granules were elliptical and had central hila. Similar morphology in *A. fortunei*, maize, potato, and pea starches has been reported in previous literature [1,23,26]. *A. fortunei* starch showed unimodal size distribution, and sizes ranged from 3 to 30 μm. However, maize, potato, and pea starches had bimodal size distribution, and the volume percentages of small granules were 8.1%, 5.8%, and 6.2%, respectively. The sizes of small and large granules ranged from 0.4 to 3 μm and from 6 to 40 μm in maize starch, from 0.6 to 6 μm and from 10 to 100 μm in potato starch, and from 0.6 to 6 μm and from 10 to 70 μm in pea starch, respectively. *A. fortunei* starch had the smallest granule size, and potato starch had the largest granule size among four starches (Table 1). Similar granule sizes in *A. fortunei*, maize, potato, and pea starches have been reported [1,23,26]. The morphology, granule size, and hilum position of starch is mainly attributed to the botany origin [27].

### 2.3. Iodine Absorption Spectrum and Apparent Amylose Content of Starch

**Figure 2:**
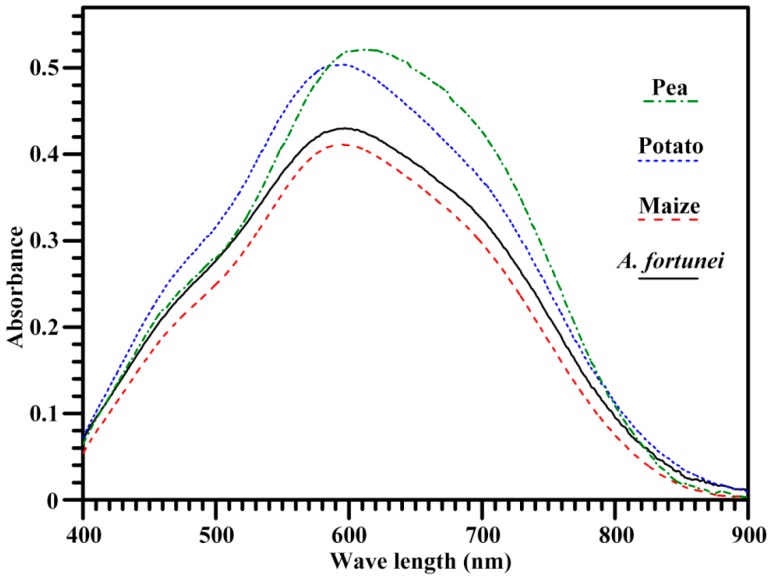
Spectrum of iodine absorbance of starch.

The absorbance spectrum of starch-iodine complex is shown in Figure 2, and its derived maximum absorption wavelength (λ_max_), iodine blue value (BV, absorbance at 680 nm), and apparent amylose content (AAC) are presented in Table 2. The λ_max_ can reflect the average chain length and polymerization degree of amylopectin and amylose, the BV is related to the affinity of starch and iodine, and the AAC indicates the absorbance of iodine by amylopectin longer branch-chains and amylose [28]. *A. fortunei* and potato starches had similar λ_max_, which was significantly higher than that of maize starch and lower than that of pea starch. Normally, AAC is an important parameter in determining the properties and applications of starch [7,21]. The AAC in A. *fortunei* starch was significantly higher than in maize starch and lower than in potato and pea starches. The AACs of maize and potato starches determined by iodine adsorption method are 31% and 43%, respectively [26], and were similar to the present results. However, *A. americana* starch has about 32% amylose determined using an iodine potentiometric autotitrator [29], legume starch has amylose content ranging from 17% to 52% [9], and tuber starch has amylose content ranging from 26% to 45% [8]. The difference in amylose content of different starches might result from the different species, varieties, growing environments, and amylose measuring methods [1,9,29,30].

### 2.4. Crystalline Structure of Starch

**Figure 3:**
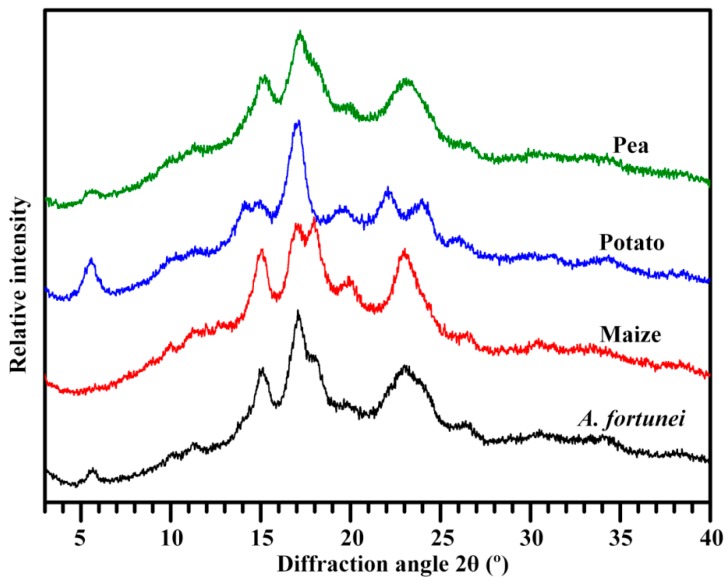
XRD pattern of starch.

The XRD patterns of starches are shown in Figure 3. Maize starch had strong diffraction peaks at about 15° and 23° 2θ and an unresolved doublet at 17° and 18° 2θ, exhibiting a typical A-type XRD pattern. Potato starch showed characteristic peaks at 5.6°, 15°, 17°, 22°, and 23° 2θ, displaying a typical B-type XRD pattern. Compared with maize and potato starches, pea starch had peak at about 5.6° and 23° 2θ, which are the characteristic peak of B-type and A-type starch, respectively, indicating that pea starch contained A- and B-type allomorphs and was C-type starch. The XRD pattern of *A. fortunei* starch was similar to that of pea starch, except that the shoulder peak at 18° 2θ was more pronounced in *A. fortunei* starch, exhibiting a C_A_-type starch in *A. fortunei*. Usually, normal cereal seeds have A-type starch, tuber crops have B-type starch, and legume seeds and some plant rhizomes have C-type starch [1,2,14,15]. However, the A-, C-, and B-type starches have also been reported in root, tuber, and legume crops [5,6]. The C-type starch is detected in root tubers of *A. americana* and *fortunei* [23,26]. The environment, especially growth temperature, has important effects on the crystalline structure of starch [1,4,30]. In root tuber of sweet potato, the low temperature forms B-type crystallinity and the high temperature forms A-type crystallinity; therefore, the proportion of A- and B-type allomorphs in C-type starch is affected by growing temperature, resulting in C_A_-, C_C_-, and C_B_-type starch [30]. The relative crystallinity was the highest in *A. fortunei* starch and the lowest in pea starch (Table 2).

### 2.5. Short-Range Ordered Structure of Starch

**Figure 4:**
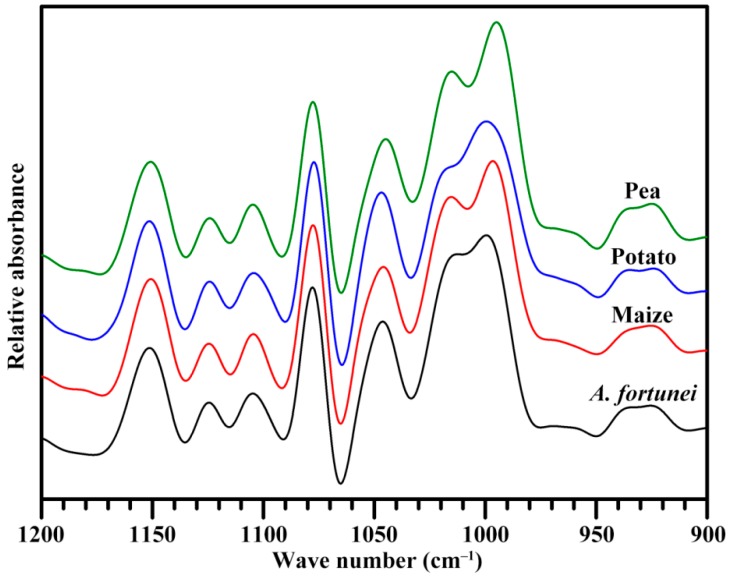
ATR-FTIR spectrum of starch.

The short-range order of starch reflects double helical order and can be measured by Fourier transform infrared (FTIR) spectrometer. The attenuated total reflectance (ATR)-FTIR spectrum is usually used to analyze the short-range ordered structure in external region of starch granule [31]. The Figure 4 shows the deconvoluted ATR-FTIR spectra of starches. The significant difference was seen at band of 1022 cm^−1^ among four starches, which is associated with amorphous region of starch. The absorbance at 1045 cm^−1^ is relative to the ordered/crystalline region of starch. The absorbance ratio of 1045/1022 cm^−1^ can show the ordered degree of starch, and that of 1022/995 cm^−1^ reflects the proportion of amorphous to ordered carbohydrate structure in starch [31]. The IR ratios of 1045/1022 and 1022/995 cm^−1^ are presented in Table 3. The *A. fortunei* starch had significantly different ratios of 1045/1022 and 1022/995 cm^−1^ from the other starches, indicating that it had different short-range ordered structure in starch external region. The ordered structure in starch external region has significant effects on swelling power, pasting viscosity, and hydrolysis of starch [32].

### 2.6. Lamellar Structure of Starch

**Figure 5:**
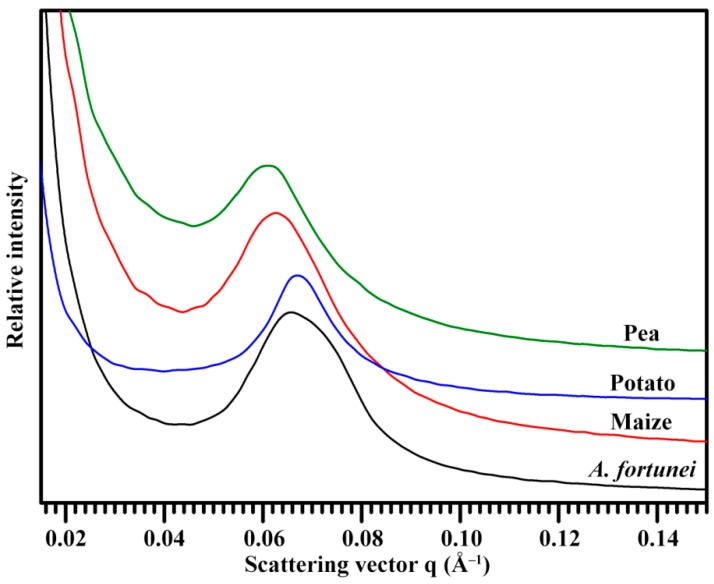
SAXS pattern of starch.

The lamellar structure of alternating amorphous and crystalline regions in starch granule can be detected by small-angle X-ray scattering (SAXS) instrument [13]. The SAXS spectra of *A. fortunei*, maize, potato, and pea starches are presented in Figure 5. All spectra were normalized to equal intensity at high q (q = 0.2 Å^−1^) to account for variations in sample concentration, causing the spectra to be at the same relative scale and therefore directly comparable [33]. The lamellar structure parameters are presented in Table 3. The lamellar peak intensity was the highest in *A. fortunei* and maize starches and the lowest in pea starch. The peak intensity reflects the degree of ordering in semicrystalline regions [34]. The main scattering peak at 0.065 Å^−1^ arises from the periodic arrangement of alternating crystalline and amorphous region and corresponds to lamellar repeat distance [13]. *A. fortunei* and potato starches had similar lamellar repeat distances, which were significantly larger than those of maize and pea starches. The lamellar structure of starch is related to plant origin but has no direct relationship with crystalline type. For the starch from the same plant origin, the amylose content is significantly negatively correlated with peak intensity and positively correlated with lamellar distance of SAXS spectrum [34,35]. Lamellar thickness and peak intensity have significant effects on swelling power, thermal properties, and hydrolysis of starch [32].

### 2.7. Thermal Properties of Starch

**Figure 6:**
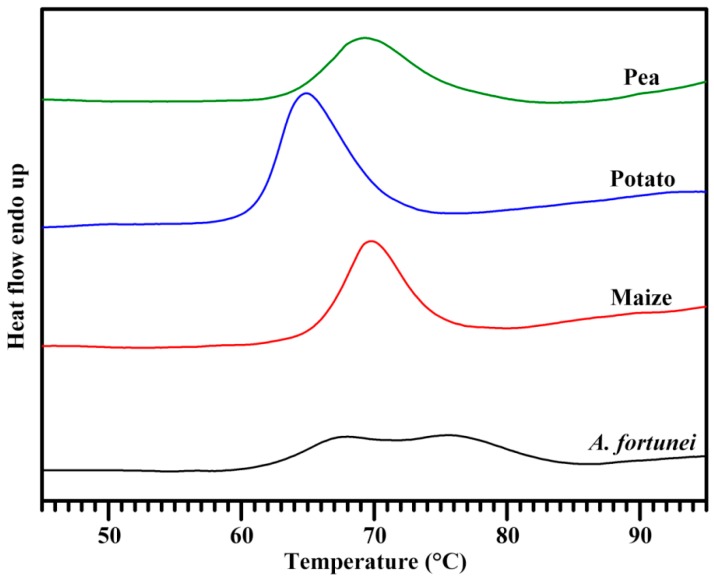
DSC thermogram of starch.

The thermal properties of *A. fortunei*, maize, potato, and pea starches are presented in Figure 6 and Table 4. The maize, potato, and pea starches had single gelatinization peak. Potato starch had significantly lower gelatinization temperature and higher gelatinization enthalpy than the other starches. However, *A. fortunei* starch had two gelatinization peaks with peak temperatures at 68.0 and 75.6 °C. The two peaks resulted in the wide gelatinization temperature range (22.1 °C). Usually, A-type crystallinity has high gelatinization temperature and B-type crystallinity has low gelatinization temperature [16,30]. C-type starch contains A- and B-type crystallinities and has wide gelatinization temperature range with single gelatinization peak in water [16,30]. When C-type starch is gelatinized in KCl solution, two gelatinization peaks are detected for the changes of gelatinization temperatures of A- and B-type crystallinities by KCl [16,30]. The two peaks of C-type starch in water have been reported in starches of A. *americana* and *fortunei* [23,29]. For pea C-type starch, A-type crystallinity is distributed in the outer of granule, and B-type crystallinity is distributed in the inner of granule [16]. However, *A. fortunei* C-type starch contains A-type starch granules and B-type starch granules [23]. The different allomorph distributions in *A. fortunei* and pea starches might result in different gelatinization properties.

### 2.8. Swelling Power and Water Solubility of Starch

**Figure 7:**
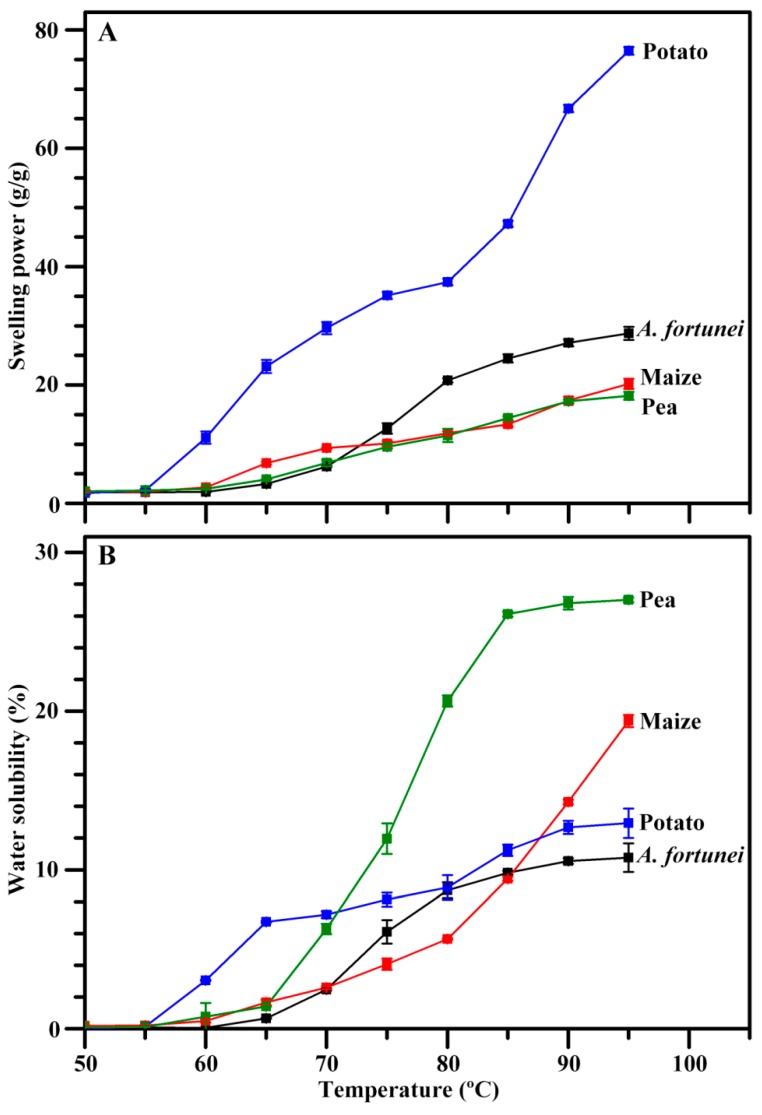
Swelling power (**A**) and water solubility (**B**) of starch.

The swelling power and water solubility of starch at different temperatures are shown in Figure 7. The swelling power and water solubility of potato starch increased after 60 °C, and those of *A. fortunei*, maize, and pea starches increased after 70 °C. After 80 °C, potato starch had the highest swelling power, maize and pea starches had the lowest swelling power, and pea starch had the highest water solubility. The swelling power and water solubility reflect the water-holding capacity and dissolution degree of starch during heating, respectively. Granule size, starch component of amylose and amylopectin, non-starch component of protein and lipid, and amylopectin structure influence the swelling power and water solubility of starch [2,10]. The significantly different morphologies, granule sizes, apparent amylose contents, and crystalline types among *A. fortunei*, maize, potato, and pea starches might account for their different swelling powers and water solubilities.

### 2.9. Pasting Properties of Starch

Pasting properties of starches, an important functional property determining the quality and utilization of starch in food industry, were measured with a rapid visco analyzer (RVA). The Table 5 shows the pasting property parameters. Peak viscosity indicates the bind ability of starch and water by hydrogen bonds, and final viscosity reflects the stability of swelling granule. Breakdown viscosity is negatively relative with the pasting resistance of starch to heat, and setback viscosity shows the tendency of starch paste to retrogradation. Pasting temperature can reflect the energy cost required during cooking [4,36]. The pasting properties of starches are affected by granule morphology, size, amylose content, crystalline structure, and swelling power [37]. Significantly different pasting properties were detected in the four starches. Potato starch had the highest peak, hot, final, and breakdown viscosities and the lowest pasting time and temperature among the four starches, which might be due to its large granule size (Table 1), low gelatinization temperature (Table 4), and high swelling power (Figure 7). *A. fortunei* starch had significantly higher pasting viscosities and lower pasting times and temperatures than maize and pea starches, which might be due to its components of A-type starch granules and B-type starch granules.

### 2.10. Digestion Properties of Starch

Native, gelatinized, and retrograded starches were digested by both porcine pancreatic α-amylase (PPA) and *Aspergillus niger* amyloglucosidase (AAG), and the released glucose was converted to the degraded starch. The starch fractions are classified into rapidly digestible starch (RDS), slowly digestible starch (SDS), and resistant starch (RS), which are digested within 20 min, between 20 and 120 min, and after 120 min, respectively [38]. The RDS, SDS, and RS contents in native, gelatinized, and retrograded starches are presented in Table 6. For native starch, *A. fortunei*, maize, and pea starches had similar RDS and were degraded faster than potato starch, and the RS of *A. fortunei* starch was significantly higher than that of maize and pea starches and lower than that of potato starch. The different digestion properties of four native starches might be affected by granule morphology and size, starch components, and crystalline structure [39]. Gelatinization destroys the granule morphology and crystalline structure through disturbing the inter- and intra-molecular hydrogen bonds of starch chains, which can cause the starch to be degraded easily. When the gelatinized starch retrogrades, the amylopectins can recrystallize to form the crystallites, and the amylose chains can associate to form the double helices structure, causing the starch to degrade more slowly than gelatinized starch [40]. *A. fortunei*, maize, potato, and pea had similar RDSs in their gelatinized and retrograded starches, but RS of gelatinized and retrograded starches was significantly lower in *A. fortunei* and pea than in maize and potato (Table 6). The difference in digestion properties of gelatinized and retrograded starches among *A. fortunei*, maize, potato, and pea might result from the difference of apparent amylose content and amylopectin structure [28].

### 2.11. Cluster Analysis of Starch

**Figure 8:**
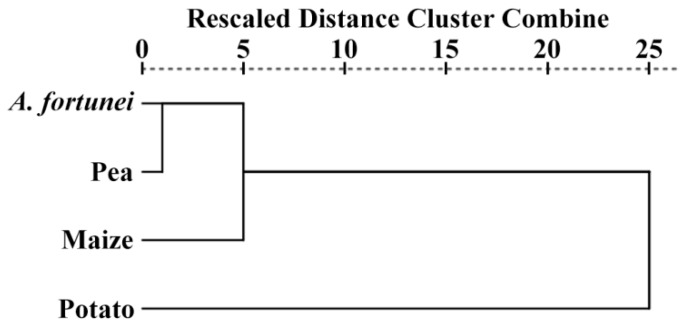
Dendrogram generated by hierarchical cluster analysis based on structural and functional property parameters of starches.

In order to compare the properties of starches from *A. fortunei*, maize, potato, and pea, the hierarchical cluster analysis was conducted based on the structural and functional property parameters including volume-weighted mean diameter, AAC, relative crystallinity, ordered and lamellar parameters, gelatinization enthalpy, pasting viscosities, and digestion properties (Figure 8). The dissimilarity between samples can be evaluated by horizontal distance that separates them. The dendrogram consisted of two major clusters. On the basis of similarities and differences in all of the property parameters, potato starch was separated from the other three starches at a linkage distance of 25. As for the remaining three starches, there were two groups at a linkage distance of approximately 5.0, and they were maize starch, *A. fortunei* starch, and pea starch. *A. fortunei* and pea starches were separated from each other by approximately 1.0. These results indicated that *A. fortunei* and pea starches showed similar physicochemical properties and were more related to maize starch than potato starch.

## 3. Materials and Methods

### 3.1. Plant. Materials

Fresh root tubers of *A. fortunei* were obtained from the natural food market (Heze City, China). Potato fresh tubers and pea mature seeds were obtained from the natural food market (Yangzhou City, China). Normal maize starch (S4126) was purchased from Sigma-Aldrich (Darmstadt, Germany).

### 3.2. Determination of Starch and Soluble Sugar Content in Root Tuber

The fresh root tubers of *A. fortunei* were washed and sliced into pieces. The samples were freeze-dried and ground extensively through 100-mesh sieve to obtain the flour. The soluble sugar in flour was extracted with 80% (*v*/*v*) ethanol, and the starch in flour was hydrolyzed into soluble sugar using 4.6 M HClO_4_. The extracted and hydrolyzed soluble sugar were measured using anthrone-H_2_SO_4_ method and converted to the contents of soluble sugar and starch in dry root tuber following the method of Gao et al. [11].

### 3.3. Starch Isolation

Starches were isolated from *A. fortunei* root tuber, potato tuber, and pea seeds following the method of Fan et al. [23], with some modifications. Briefly, the dry pea seeds were softened in water overnight at 4 °C, and the *A. fortunei* root tubers and potato tubers were washed and chopped into pieces. The samples were homogenized in water using a home blender (JYL-C93T, Joyoung, Suzhou, Jiangsu, China) and squeezed through 4 layers of cheesecloth. The starch-slurry was filtered using 100- and 200-mesh sieves (Yueyang, Taizhou, Zhejiang, China). After standing about 5 h, the precipitated starch was washed 3 times using 0.2% NaOH to remove the surface protein from starch granules. The starch was washed 3 times with water and 2 times with anhydrous ethanol, dried at 40 °C, and ground through 100-mesh sieve.

### 3.4. Morphology Observation and Granule Size Analysis

The 1% (*w*/*v*) starch suspension in 50% glycerol was observed and photographed under a polarized light microscope (BX53, Olympus, Tokyo, Japan) under normal and polarized light. The size distribution of starch granules was measured using a laser diffraction instrument (Mastersizer 2000, Malvern, Worcestershire, UK) following the method of Cai et al. [21].

### 3.5. Measurements of Iodine Absorption Spectrum and AAC

The iodine absorption spectrum of starch and AAC were determined following the method of Lin et al. [28]. Briefly, starch was dissolved in urea dimethyl sulphoxide solution and stained with iodine solution. The iodine absorption spectrum was scanned from 400 to 900 nm with a spectrophotometer (Ultrospec 6300 pro, Amersham Bioscience, Cambridge, UK). AAC was evaluated from absorbance at 620 nm.

### 3.6. Crystalline Structure Analysis

Dry starch was first wetted for 1 week in 75% humidity. The starch was scanned from 3° to 40° 2θ with a step size of 0.02° under an X-ray powder diffractometer (D8, Bruker, Karlsruhe, Germany). The relative crystallinity was calculated following the method of Wei et al. [18].

### 3.7. Short-Range Ordered Structure Analysis

The starch-water slurry was analyzed using a FTIR spectrometer (7000, Varian, Santa Clara, CA, USA) with a DTGS detector equipped with an ATR cell following the method of Wei et al. [18]. Original spectrum was corrected by subtraction of the baseline in the region from 1200 to 800 cm^−1^ before deconvolution. For deconvolution, the assumed line shape was Lorentzian with a half-width of 19 cm^−1^ and a resolution enhancement factor of 1.9.

### 3.8. Lamellar Structure Analysis

The starch-water slurry was kept in sealed cell and analyzed using a SAXS instrument (NanoStar, Bruker, Karlsruhe, Germany) equipped with Vantec 2000 detector and pin-hole collimation for point focus geometry. The test condition setting and lamellar parameter analysis were described previously by Cai et al. [19].

### 3.9. Measurement of Swelling Power and Water Solubility

The swelling power and water solubility of starch were measured from 50 to 95 °C at an interval of 5 °C following the method of Lin et al. [7]. Briefly, 2% (*w*/*v*) starch-water slurry was heated in a ThermoMixer C (Eppendorf, Hamburg, Germany) for 30 min, cooled to room temperature, and centrifuged (8000× *g*, 10 min). The supernatant was removed for measuring the soluble carbohydrate using anthrone-H_2_SO_4_ method to calculate the water solubility, and the precipitate was weighed to calculate the swelling power.

### 3.10. Measurement of Thermal Properties

The five milligram of starch and 15 μL of water were mixed and sealed in an aluminium pan (Netzsch, Selb, Germany), held at 4 °C overnight, and equilibrated for 2 h at room temperature. The sample was heated from 25 to 130 °C at a rate of 10 °C/min using a differential scanning calorimeter (200-F3, Netzsch, Selb, Germany).

### 3.11. Measurement of Pasting Properties

The 7% (*w*/*w*) starch-water slurry was heated and cooled using a rapid visco analyzer (3D, Newport Scientific, Warriewood, NSW, Australia). The temperature program included holding for 1 min at 50 °C, heating at 12 °C/min to 95 °C, holding for 2.5 min at 95 °C, cooling at 12 °C/min to 50 °C, and holding for 1.4 min at 50 °C.

### 3.12. Measurement of Digestion Properties

The native, gelatinized, and retrograded starches were prepared and degraded by both PPA (A3176, Sigma Aldrich, St. Louis, MO, USA) and AAG (E-AMGDF, Megazyme, Bray, Ireland) following the method of Lin et al. [28]. Briefly, the 10 mg of starch was incubated in 2 mL of enzyme solution (20 mM sodium phosphate buffer, pH 6.0, 6.7 mM NaCl, 0.01% NaN_3_, 2.5 mM CaCl_2_, 4 U PPA, and 4 U AAG) at 37 °C using a ThermoMixer C (Eppendorf, Hamburg, Germany) with shaking at 1000 rpm. The hydrolysis was terminated by adding 240 mL of 0.1 M HCl and 2 mL of 50% ethanol, and then centrifuged (4 °C, 8000× *g*, 5 min). The glucose in the supernatant was measured using a glucose assay kit (K-GLUC, Megazyme, Bray, Ireland).

### 3.13. Statistical Analysis

The data reported in all the tables were means ± standard deviation. The one-way analysis of variance by Tukey’s test was evaluated using the SPSS 19.0 Statistical Software Program (IBM Company, Chicago, IL, USA). Differences were considered statistically significant if *p*-values were less 0.05. Hierarchical cluster analysis was employed using between-groups linkage as the cluster method and Pearson correlation as the interval measure.

## 4. Conclusions

The root tuber of *A. fortunei* is an important starch resource. Starch exhibited spherical, polygonal, and ellipsoidal granules with central hila and had a unimodal size distribution that ranged from 3 to 30 μm. Starch had 35% AAC and exhibited C_A_-type crystallinity. Starch had two gelatinization peaks in water. The swelling power of *A. fortunei* starch was significantly lower than that of potato starch and higher than that of maize and pea starches at 95 °C, but its water solubility was the lowest among the four starches. The peak, hot, breakdown, and final viscosities of *A. fortunei* starch were significantly lower than those of potato starch and higher than those of maize and potato starches, but its setback viscosity was the highest among the four starches. The RDS of native starches from *A. fortunei*, maize, and pea was similar but significantly higher than that of potato starch. The RS of gelatinized and retrograded starches from *A. fortunei* and pea was similar but significantly lower than that of maize and potato starches. This study could provide important information for the utilization of root tuber starch of *A. fortunei*.

## Figures and Tables

**Table 1:** Granule sizes of starch.

Starches	d(0.1) (μm)	d(0.5) (μm)	d(0.9) (μm)	D[4,3] (μm)
*A. fortunei*	5.403 ± 0.002 ^a^	8.947 ± 0.002 ^a^	14.760 ± 0.004 ^a^	9.584 ± 0.002 ^a^
Maize	7.519 ± 0.031 ^b^	13.767 ± 0.078 ^b^	21.305 ± 0.173 ^b^	13.895 ± 0.086 ^b^
Potato	16.317 ± 0.010 ^c^	34.420 ± 0.004 ^d^	62.406 ± 0.036 ^d^	36.778 ± 0.006 ^d^
Pea	16.255 ± 0.078 ^c^	27.518 ± 0.169 ^c^	41.917 ± 0.359 ^c^	27.922 ± 0.204 ^c^

The d(0.1), d(0.5), and d(0.9) are the granule sizes at which 10, 50, and 90% of all the granules by volume are smaller, respectively. The D[4,3] is the volume-weighted mean diameter. Data are means ± standard deviations, *n* = 3. Different superscript letters (from ^a^ to ^d^) in the same column indicate significantly difference at *p* < 0.05.

**Table 2:** Maximum absorption wavelength (λ_max_), iodine blue value (BV), apparent amylose content (AAC), and relative crystallinity (RC) of starch.

Starches	λ_max_ (nm)	BV	AAC (%)	RC (%)
*A. fortunei*	597.3 ± 0.6 ^b^	0.355 ± 0.007 ^b^	35.0 ± 0.6 ^b^	25.9 ± 0.1 ^c^
Maize	594.3 ± 0.6 ^a^	0.328 ± 0.003 ^a^	30.9 ± 0.3 ^a^	20.0 ± 0.9 ^b^
Potato	598.3 ± 0.6 ^b^	0.403 ± 0.008 ^c^	41.0 ± 0.4 ^c^	20.0 ± 0.6 ^b^
Pea	613.3 ± 0.6 ^c^	0.460 ± 0.006 ^d^	47.0 ± 0.3 ^d^	16.9 ± 0.1 ^a^

Data are means ± standard deviations, *n* = 3. Different superscript letters (from ^a^ to ^d^) in the same column indicate significantly difference at *p* < 0.05.

**Table 3:** Parameters of short-range ordered structure and lamellar structure of starch.

Starches	Ordered Structure Parameters		Lamellar Structure Parameters		
	1045/1022 cm^−^^1^	1022/995 cm^−^^1^	I_max_ (Counts)	S_max_ (Å^−^^1^)	D (nm)
*A. fortunei*	0.648 ± 0.004 ^b^	0.883 ± 0.018 ^c^	215.3 ± 0.7 ^c^	0.066 ± 0.001 ^b^	9.60 ± 0.14 ^a^
Maize	0.623 ± 0.009 ^a^	0.837 ± 0.016 ^b^	217.7 ± 6.5 ^c^	0.063 ± 0.001 ^a^	10.05 ± 0.07 ^b^
Potato	0.816 ± 0.010 ^c^	0.763 ± 0.011 ^a^	170.1 ± 0.4 ^b^	0.067 ± 0.001 ^b^	9.40 ± 0.01 ^a^
Pea	0.623 ± 0.008 ^a^	0.781 ± 0.005 ^a^	154.6 ± 3.8 ^a^	0.061 ± 0.001 ^a^	10.30 ± 0.01 ^b^

I_max_: lamellar peak intensity; S_max_: lamellar peak position; D: lamellar thickness. Data are means ± standard deviations, *n* = 2. Different superscript letters (from ^a^ to ^c^) in the same column indicate significantly difference at *p* < 0.05.

**Table 4:** Thermal parameters of starch.

Starches	To (°C)	Tp1 (°C)	Tp2 (°C)	Tc (°C)	ΔT (°C)	ΔH (J/g)
*A. fortunei*	61.8 ± 0.3 ^b^	68.0 ± 0.3 ^b^	75.6 ± 0.1 ^c^	83.9 ± 0.3 ^d^	22.1 ± 0.4 ^d^	10.2 ± 0.9 ^a^
Maize	65.8 ± 0.2 ^d^	ND	70.0 ± 0.1 ^b^	74.7 ± 0.1 ^b^	9.0 ± 0.1 ^a^	10.5 ± 0.4 ^a^
Potato	61.0 ± 0.1 ^a^	64.8 ± 0.1 ^a^	ND	70.7 ± 0.3 ^a^	9.6 ± 0.3 ^b^	14.9 ± 0.3 ^b^
Pea	63.6 ± 0.1 ^c^	ND	69.3 ± 0.1 ^a^	76.6 ± 0.2 ^c^	13.0 ± 0.2 ^c^	9.5 ± 0.6 ^a^

To: gelatinization onset temperature; Tp1: peak temperature of the first gelatinization peak; Tp2: peak temperature of the second gelatinization peak; Tc: gelatinization conclusion temperature; ΔT: gelatinization temperature range (Tc–To); ΔH: gelatinization enthalpy; ND: not detected. Data are means ± standard deviations, *n* = 3. Different superscript letters (from ^a^ to ^d^) in the same column indicate significantly difference at *p* < 0.05.

**Table 5:** Pasting parameters of starch.

Starches	PV (mPa s)	HV (mPa s)	BV (mPa s)	FV (mPa s)	SV (mPa s)	P_Time_ (min)	P_Temp_ (°C)
*A. fortunei*	1689 ± 16 ^c^	1420 ± 29 ^c^	269 ± 15 ^b^	2103 ± 37 ^c^	683 ± 8 ^d^	5.04 ± 0.04 ^b^	80.80 ± 0.48 ^b^
Maize	954 ± 15 ^b^	770 ± 9 ^b^	184 ± 6 ^a^	887 ± 9 ^b^	117 ± 1 ^a^	5.73 ± 0.01 ^c^	91.88 ± 0.03 ^c^
Potato	7860 ± 30 ^d^	3285 ± 32 ^d^	4574 ± 50 ^c^	3682 ± 18 ^d^	396 ± 14 ^c^	3.89 ± 0.04 ^a^	68.67 ± 0.46 ^a^
Pea	580 ± 23 ^a^	438 ± 21 ^a^	141 ± 4 ^a^	795 ± 21 ^a^	357 ± 9 ^b^	7.00 ± 0.01 ^d^	94.85 ± 0.76 ^d^

PV: peak viscosity; HV: hot viscosity; BV: breakdown viscosity (PV−HV); FV: final viscosity; SV: setback viscosity (FV−HV); P_Time_: peak time; P_Temp_: pasting temperature. Data are means ± standard deviations, *n* = 3. Different superscript letters (from ^a^ to ^d^) in the same column indicate significantly difference at *p* < 0.05.

**Table 6:** Digestion properties of starch.

Starches	Components	*A. fortunei*	Maize	Potato	Pea
Native starch	RDS (%)	6.04 ± 0.62 ^b^	6.45 ± 0.03 ^b^	2.27 ± 0.01 ^a^	6.20 ± 0.13 ^b^
	SDS (%)	10.96 ± 0.41 ^b^	22.98 ± 0.65 ^d^	6.33 ± 0.23 ^a^	20.23 ± 0.29 ^c^
	RS (%)	83.00 ± 0.22 ^c^	70.56 ± 0.68 ^a^	91.40 ± 0.24 ^d^	73.57 ± 0.42 ^b^
Gelatinized starch	RDS (%)	83.16 ± 1.33 ^a^	81.59 ± 0.57 ^a^	84.14 ± 0.62 ^a^	83.66 ± 1.23 ^a^
	SDS (%)	15.23 ± 0.12 ^d^	7.97 ± 0.23 ^a^	8.49 ± 0.14 ^b^	14.55 ± 0.33 ^c^
	RS (%)	1.61 ± 1.28 ^a^	10.44 ± 0.44 ^c^	7.37 ± 0.73 ^b^	1.79 ± 1.41 ^a^
Retrograded starch	RDS (%)	78.13 ± 3.90 ^a^	78.72 ± 0.72 ^a^	80.15 ± 0.45 ^a^	78.49 ± 1.26 ^a^
	SDS (%)	17.88 ± 2.27 ^b^	7.99 ± 0.48 ^a^	8.29 ± 0.21 ^a^	17.80 ± 0.38 ^b^
	RS (%)	3.99 ± 1.64 ^a^	13.29 ± 0.26 ^b^	11.57 ± 0.27 ^b^	3.72 ± 0.96 ^a^

RDS: rapidly digestible starch; SDS: slowly digestible starch; RS: resistant starch. Data are means ± standard deviations, *n* = 3. Different superscript letters (from ^a^ to ^d^) in the row column indicate significantly difference at *p* < 0.05.
